# Spatio-temporal epidemiology of the tuberculosis incidence rate in Iran 2008 to 2018

**DOI:** 10.1186/s12889-021-11157-1

**Published:** 2021-06-07

**Authors:** Behzad Kiani, Amene Raouf Rahmati, Robert Bergquist, Soheil Hashtarkhani, Neda Firouraghi, Nasser Bagheri, Elham Moghaddas, Alireza Mohammadi

**Affiliations:** 1 Department of Medical Informatics, School of Medicine, Mashhad University of Medical Sciences, Mashhad, Iran; 2 Department of Parasitology and Mycology, School of Medicine, Mashhad University of Medical Sciences, Mashhad, Iran; 3 Ingerod, Brastad, Lysekil, Sweden; 4 formerly with the UNICEF/UNDP/World Bank/WHO Special Programme for Research and Training in Tropical Diseases, World Health Organization, Geneva, Switzerland; 5 Center for Mental Health Research College of Health and Medicine, Australian National University, Canberra, Australian Capital Territory Australia; 6 Department of Geography and Urban Planning, Faculty of Social Sciences, University of Mohaghegh Ardabili, Ardabil, Iran

**Keywords:** Iran, Geographical information systems, SaTScan, Spatial analysis, Spatial scan spatiotemporal, Tuberculosis

## Abstract

**Background:**

Effective reduction of tuberculosis (TB) requires information on the distribution of TB incidence rate across time and location. This study aims to identify the spatio-temporal pattern of TB incidence rate in Iran between 2008 and 2018.

**Methods:**

This cross-sectional study was conducted on aggregated TB data (50,500 patients) at the provincial level provided by the Ministry of Health in Iran between 2008 and 2018. The Anselin Local Moran’s *I* and Getis-Ord Gi* were performed to identify the spatial variations of the disease. Furthermore, spatial scan statistic was employed for purely temporal and spatio-temporal analyses. In all instances, the null hypothesis of no clusters was rejected at *p* ≤ 0.05.

**Results:**

The overall incidence rate of TB decreased from 13.46 per 100,000 (95% CI: 13.19–13.73) in 2008 to 10.88 per 100,000 (95% CI: 10.65–11.11) in 2018. The highest incidence rate of TB was observed in southeast and northeast of Iran for the whole study period. Additionally, spatial cluster analysis discovered Khuzestan Province, in the West of the country, having significantly higher rates than neighbouring provinces in terms of both total TB and smear-positive pulmonary TB (SPPTB). Purely temporal analysis showed that high-rate and low-rate clusters were predominantly distributed in the time periods 2010–2014 and 2017–2018. Spatio-temporal results showed that the statistically significant clusters were mainly distributed from centre to the east during the study period. Some high-trend TB and SPPTB statistically significant clusters were found.

**Conclusion:**

The results provided an overview of the latest TB spatio-temporal status In Iran and identified decreasing trends of TB in the 2008–2018 period. Despite the decreasing incidence rate, there is still need for screening, and targeting of preventive interventions, especially in high-risk areas. Knowledge of the spatio-temporal pattern of TB can be useful for policy development as the information regarding the high-risk areas would contribute to the selection of areas needed to be targeted for the expansion of health facilities.

**Supplementary Information:**

The online version contains supplementary material available at 10.1186/s12889-021-11157-1.

## Background

Tuberculosis (TB), an infectious disease caused by *Mycobacterium tuberculosis*, continues to infect many people in spite of global attempts to control the disease. According to the World Health Organization (WHO), 10 million people suffer from TB across the world in 2018, incurring 1.5 million deaths [1]. However, WHO statistics show that only 61% of TB patients have been detected up to 2015 [2]. To reach the ultimate goal of eliminating TB by 2050 (defined as ≤1 case per 1 million persons) [3], the identification of high-risk areas is a crucial need for each country.

TB remains a major public health problem in developing countries such as Iran. In this country, the TB incidence rate stood at 16 persons per 100,000 in 2015, declining to 14 per 100,000 people in 2016 and 2017 [4–6]. Patients with sputum smear-negative TB (SSNTB) are less infectious than patients with sputum smear-positive TB (SSPTB) [7]. A new detected SSPTB patient costs US$ 1409 for the Iranian health system [8]. A systematic review and meta-analysis in 2019 showed that health care workers residing in the northern and the western regions of the country had the highest prevalence of latent TB, i.e. carriers in good condition but with very small numbers of bacteria [9]. In these patients, symptoms are not apparent as the infection is kept under control by the body’s immune system [10]. Iran shares extensive borders with high TB-burden countries, such as Azerbaijan, Turkmenistan, Armenia, Pakistan, Afghanistan and Iraq [11–14]. Increased immigration and travelling to Iran from neighbouring countries [15–18] cause major obstacles for TB control [19]. As the distribution of the disease varies in different parts of the country [20], efficient screening programmes by identifying the high-risk areas are required.

Identification of spatial and spatio-temporal patterns of diseases can be done by Geographical Information Systems (GIS) approaches [21–25], which store data by two components; spatial and non-spatial [26–29]. The former depends on geography, e.g., patient’s residence location, while the latter carries other information, e.g., patient data such as age, gender, etc. GIS enables visualization and monitoring of infectious diseases by conducting spatio-temporal analyses that combine spatial and non-spatial data as well as accounting for the time componenent [30]. Spatio-temporal pattern analysis identifies data distributions and patterns in the context of both space and time [31].

A TB study in Pakistan [32] conducted by spatial scan statistics identified spatio-temporal clustering with high-risk clusters across the country during 2015–2019, particularly in the northern and western parts of the study area. Another study, assessing spatial and temporal pattern of TB in the 75 municipalities in the Northeast of Brazil during 2001 to 2016 by empirical Bayesian and Moran statistic [23], noted an increased trend of TB in patients under 40 years of age. Masabarakiza et al. [33] used GIS to analyse the distribution of the TB incidence in Burundi during the 2009–2017 period, and reported that the eastern parts of Burundi had experienced a relatively low incidence rates of TB compared to other parts of the country. A retrospective study from Western Kenya [25], analyzing the spatial distribution of 23,374 TB cases from 2012 to 2015, noted that the TB incidence varied from 638.0 to 121.4 persons per 100,000 at the small-area level. Using spatial clustering analysis according to Moran and Getis-Ord Gi* statistic helped them to identify 16 districts with high TB incidence [34]. A spatial and spatio-temporal study of TB in Southern Ethiopia conducted from 2007 to 2016 [26] revealed strong variation of TB from 70.4 to 155.3 persons per 100,000 population. In this study, based on Global Moran’s *I*, Getis-Ord and scan statistics, eleven purely spatial and three spatio-temporal clusters were identified [35]. A TB study in China [36], carried out in the 2009–2016 period, revealed some spatial, temporal, and spatio-temporal clustered areas based on scan statistics. Another study in China by Wubuli et al. [37] used spatial autocorrelation analysis based on Moran’s *I* and local Getis-Ord statistics and reported the presence of a non-random geographical distribution for pulmonary TB incidence between 2005 and 2013. The high-risk areas consistently located in the south-western regions and low-risk ones in the north-central regions.

With respect to Iran, a few studies assessed the spatial patterns of TB incidence in some provinces, e.g., a spatio-temporal, cross-sectional mapping of TB incidence, conducted during 2007–2012 in the Northeast that assessed 155 TB cases and identified a downward incidence trend [38]. An ecological study in south-western Iran described the spatial distribution of TB and the Human Immunodeficiency Virus (HIV) from 2007 to 2011. This study, based on global and local Moran statistics, identified a spatial pattern distribution of TB and HIV [39]. Another study was conducted in northern Iran on 2444 TB patients in the period 1999–2008. The Moran’s *I* statistic revealed spatial clustering of TB incidence [40]. Only one study in Iran has analysed the geographical distribution of TB cases at the provincial level with a countryside scale. It covered the period 2013 to 2017 but the results were more descriptive than spatio-temporal [41].

The Iran Ministry of Health and Medical Education (MOHME) plans to reach TB eradication for the whole country by 2050 [42]. To that end, it holds periodic training workshops about transmission and prevention of the disease. In this context, we undertook an exploration of the spatial, temporal and spatio-temporal trend of TB at the provincial level in the whole country with the aim of contributing to the planning of efficient programmes. We felt that an evaluation of the effectiveness of national control and prevention programmes can reveal high priority provinces with regard to TB control. In addition, spatio-temporal maps can provide a retrospective view of the efficacy of intervention and control programmes. To the best of our knowledge, the present study would be the first epidemiological exploration on spatial and spatio-temporal TB clusters in the whole of Iran.

## Methods

### Study area

**Fig. 1:**
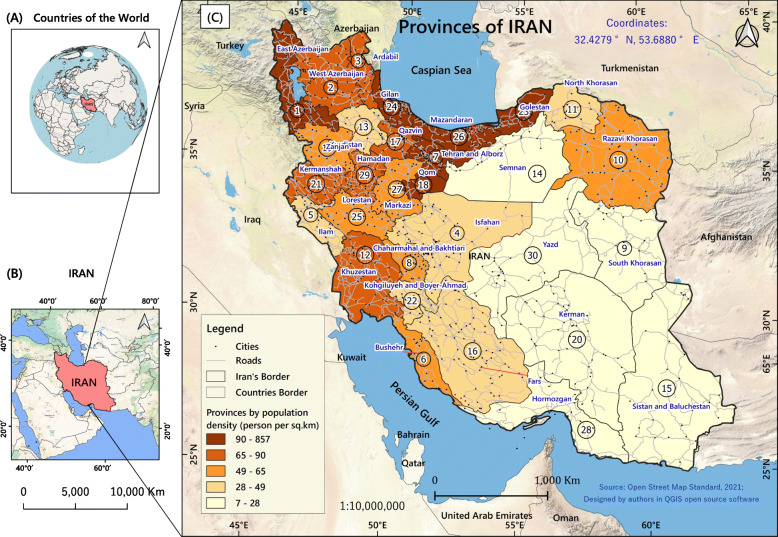
Map of the study area. Iran location (**A**) on a world map and (**B**) on map of the Middle East; (**C**) indicates the 30 provinces of Iran. The colours from pale yellow to dark brown indicate the changing population density per km^2^ from low to high. The numbers in each of the polygons are identification codes for the study locations (provinces). The figure has been created by free QGIS software

The study covered 50,500 TB patients at the provincial level across all of Iran (Fig. 1). Iran is the world’s 18th most populous country with 82 million inhabitants. Its territory spans 1,648,195 km^2^ making it the second-largest country in the Middle-East and the 17th largest in the world [43]. There used to be 30 provinces in the country, but increased to 31 after the province of Tehran was divided into two, Tehran and Alborz, in 2010. However, as the study took place from 2008 to 2018, we kept the old number of 30 provinces for the spatial analyses.

### TB definition and diagnosis

All suspected individuals were detected by microscopy of Ziehl-Neelsen stained sputum smears. All non-pulmonary TB patients were recognized by pathological methods in Iran.

### Data sources

Three different data sources were used. First, the TB data was obtained through the MOHME (supplementary file 1); The TB data were recorded according to the official Persian calendar where the year starts at the spring equinox, which corresponds to around 20 March in the Gregorian calendar used in the West. Second, the provincial division in Iran was obtained through the Mapping Organization of the country. Finally, the population data was obtained via the statistical centre of Iran [44]. The country conducts population census every 5 years, however for other years, the statistical centre of Iran estimates the population for each province and releases the data separately. In our study, the population census data were available for the years 2012 and 2017, but we used the statistical centre data for the other years [45] (supplementary file 1).

### Spatial analyses

#### Descriptive spatial analysis

The manual classification was used for producing descriptive maps. In the classification of rates (Fig. 3), the lowest and highest values of the period were considered as lower and higher classes. Other classes were classified at rates of 5 and 10 per 100,000 people. The Wilson method was used to calculate the confidence intervals.

#### Statistical spatial analysis

Global and Local spatial statistics can be used to recognise geographical variations in the TB rate. The former ones, e.g., K-function, Cuzick Edwards, Kernel density estimation, and Global Moran’s *I*, are more sensitive to departures from the null hypothesis, which assumes that TB are randomly distributed in the area under study. Although they can identify spatial structures, they do not determine where the clusters are [46]. Local cluster statistics, on the other hand, e.g., Anselin’s Local Moran’s *I* and Getis-Ord Gi* quantify spatial autocorrelation and identify the clusters’ location. As this study aimed to detect the location of potential clusters, Anselin’s local Moran’s *I* and Getis-Ord Gi* were used for spatial analysis. The null hypothesis regarding both of these analyses assumes that there is no association between the value observed at a location and the values observed at nearby sites.

Anselin’s Local Moran’s *I* (ALMI*)* identifies High-High clusters, Low-Low clusters, and spatial outliers (High-Low and Low-High) [47]. High-High clusters indicate areas with a high TB incidence rates surrounded by areas with similarly high TB rates, while Low-Low clusters designate areas with low TB incidence rates surrounded by areas with low rates; High-Low outliers specifies areas with high TB incidence rates surrounded by areas with low rates, while Low-High outliers indicate the opposite, i.e. low TB incidence rates surrounded by high rates [48]. ALMI calculates a z-score and *p*-value for each feature in the dataset. In this study, the ALMI results (z-scores) were considered statistically significant at a 95% confidence level (*p* <  0.05). The index is computed by eq. 1:

1 $$ {I}_i=\frac{\sum_{j=1}^n\kern0.5em {w}_{ij}\left({x}_i-\overline{x}\right)\left({x}_j-\overline{x}\right)}{\frac{1}{n}{\sum}_{i=1}^n\left({x}_i-\overline{x}\right){}^2},i\ne j $$

where *n* is the number of provinces; *x*_*i*_ and *x*_*j*_ the TB incidence rate in province *i* and *j*, respectively; $\overline{x}$ the average of the reported TB incidence rates in all provinces; and *w*_*ij*_ the spatial weight matrix corresponding to provinces *i* and *j*; and *I* the local Moran’s *I* [47–49].

The local Getis-Ord G_i_* statistic, a hotspot analysis function in ArcGIS (v.10.8, ESRI Inc., CA, USA), was used to investigate the intensity of incident TB cases, a measure of hot- or cold spot TB occurrence in Iran [50, 51]. To calculate this statistic, the value of proximity threshold was determined at 180 km for the study area using the Distance Band tool in ArcGIS. The statistical significance of a Z-score for each province was quantified through the presence of hotspots and coldspots of TB incidence relative to the hypothesis of spatial randomness [52]. The Getis-Ord G_i_* calculates a Z-score where a significant positive Z-score (Gi*) indicates a hotspot phenomenon, while a significant negative Z-score indicates a coldspot [49–51]. Provinces with a Z-scores > 1.96 at 95% confidence level (*p* <  0.05) were categorised as TB incidence hotspots. Likewise, provinces with a Z-score of <− 1.96 indicated coldspots, i.e. areas with a very low level of TB incidence. The Getis-Ord local statistic is given as eq. 2 [51, 53]:

2 $$ {G}_i^{\ast }=\frac{\sum \limits_{j=1}^n{w}_{i,j}-\overline{x}\sum \limits_{j=1}^n{w}_{i,j}}{\sqrt[S]{n\sum \limits_{j=1}^n{w}_{i,j}^2-{\left(\sum \limits_{j=1}^n{w}_{i,j}\right)}^2}} $$

where *x*_*j*_ is the TB incidence rate value for the *j*-th province. *w*_*i,j*_ the spatial weight between the *i*-th and the *j*-th province, and *n* the total number of provinces. $\overline{x}$ and *s* are the arithmetic mean and the standard deviation, respectively, of the TB rates in *n* provinces.

### Temporal and spatio-temporal analysis

To identify the temporal and spatio-temporal clustering of TB incidence rates, the spatial scan statistics, introduced during the 1960s in the field of health sciences by Naus (1965) [54], was used. Spatial and temporal extensions of the scan methods were introduced by Kulldorff from 1997 [55] and have been applied in various fields in the health domain [56, 57]. This statistic allows pre-defined spatial and temporal windows to create a moving window that considers multiple spatial and temporal scales. Depending on the availability of data, the spatial scan statistic can be used for either aggregated data, such as provincial areas, or with precise geographical coordinates, where each area contains only one person at risk [58]. Relative risk (RR), Log likehood ratio (LLR) and the Mont Carlo test, which are described in details below, constitue approaches for the interpretation of space-time analysis in scan statistics.

#### Relative risk (RR)

RR is the estimated risk within the cluster divided by the estimated risk outside the cluster, which can be mathematically calculated by eq. 3:

3 $$ RR=\frac{c/E\left[c\right]}{\left(C-c\right)/\left(\mathrm{E}\left[C\right]-\mathrm{E}\left[\mathrm{c}\right]\right)}=\frac{c/E\left[c\right]}{\left(C-c\right)/\left(C-E\left[c\right]\right)} $$

where c is the number of observed cases (TB cases in this study) within the cluster; and C the total number of cases in the dataset. Note that E [C] = C since the analysis is conditioned on the total number of cases observed [59].

#### Log likelihood ratio (LLR)

Let I be the collection of all the geographic units (provinces in this case) in study region S. Zone i consists of neighbouring provinces and can have varying shapes and sizes. Let c_i_ and n_i_ be the observed number of cases and the expected number of cases (or population) in zone i, respectively. Then C and N (eq. 4) will be the total number of cases and the total number of expected cases in S, respectively.

4 $$ C=\sum \limits_i{c}_i\ \mathrm{and}\ N=\sum \limits_i{n}_i $$

For TB incidence, a Poisson model is typically chosen. The LLR of a zone i is then given by eq. 5 [60, 61]:

5 $$ LRR(i)=\left\{{\left(\frac{c_i}{n_i}\right)}^{c_i}{\left(\frac{C-{c}_i}{N-{n}_i}\right)}^{C-{c}_i}\right\}I\left({c}_i>{n}_i\right) $$

If there is interest in scanning for ‘negative clusters’ with a lower rate than expected, the indicator function is replaced by I (c_i_ < n_i_) and if the interest is in clusters of both higher and lower rates, the indicator function is removed. It is equivalent but numerically easier to work with the logarithm, and the test statistic is given by eq. 6 that is the most likely cluster of the scanning window i ∈ I, which maximizes the LLR.

6 $$ T=\underset{i}{\max}\log \left( LR(i)\right)=\underset{i}{\max } LLR(i) $$

#### The Monte Carlo test

The test statistic is calculated for each random replication as well as for the real data set, and if the latter is among the 5 highest percentage cases, then the test is significant at the 0.05 level. If it is among the highest percentage case, the test is significant at the 0.01 level, and so on. This is called Monte Carlo hypothesis testing and was first proposed by Dwass [62]. Irrespective of the number of Monte Carlo replications chosen, the hypothesis is unbiased, resulting in a correct significance level that is neither conservative nor liberal or an estimate. The number of replications does affect the power of the test, with more replications giving a higher power [59]. The test statistics T follows approximately an extreme value distribution [63, 64], but the exact distribution is unknown, so statistical significance is evaluated using Monte Carlo hypothesis testing. This is done by creating a large number of random data sets generated under the null hypothesis that there is no cluster, and calculating the value of the test statistic for each of those random datasets. The Monte Carlo *p*-value is then calculated as r/(1 + m) where r is the rank of the test statistic from the real data set among all the random data sets and m the number of random datasets. For example, with a statistical significance level of α = 0.05, the cluster will cause a rejection of the null hypothesis if its likelihood ratio is within the highest 5% among all the maximum likelihood ratio from the one real and the m random datasets [59, 65].

#### Purely temporal cluster analysis

Purely temporal cluster analysis is a retrospective type of scan statistics that only detects time clusters in a specified time period for a specific geographic area and does not address their spatial variations or patterns [59]. SaTScan v. 9.7 includes purely temporal scan statistics. For this purpose, a purely temporal cluster analysis was performed to detect the temporal clusters of total TB and SPPTB in the time period 2008–2018. The Poisson discrete scan statistic was set as probability model to detect clusters in areas with high and low rates; the length of time aggregation was set at 1 year; to get a better understanding of the clustering tendency, the analyzes were performed with three window sizes: 25, 35 and 50%.

#### Spatio-temporal analysis

Space-time scan statistics, introduced by Martin Kulldorff [66], is the approach that the statistic inference is adjusted for multiple testing arising from many possible geographic locations and sizes of disease clusters. The method can detect spatial clusters irrespective of any predefined geographical boundries by combining any number of close locations into the same cluster in predefined time periods. This method was designed to test whether a disease is randomly distributed over space and time with the ability to repeat similar analyses [66]. In this study, space-time discrete type of scan statistics with space-time permutation probability was used to analyse the spatio-temporal clustering of total TB and SPPTB. While repeating the settings made in the purely temporal analysis, maximum spatial and temporal clustering analysis window sizes were adjusted to 25, 35 and 50% for both study area and time period. A circular shape was chosen for the spatial cluster outputs. For statistical inference, 999 Monte Carlo replications were performed. The null hypothesis of no clusters was rejected at the simulated *p* ≤ 0.05 for the primary cluster and *p =* 0.1 for the secondary clusters since the latter ones have conservative *p*-values [59].

#### Analysis of spatial variation in temporal trends

Spatial variation in temporal trends (SVTT) is relatively new approach, which identifies the clustering of geographical locations according to a common trend that is significantly different from trends in outside areas [67]. The null hypothesis is that the trends are the same, while the alternative is that they are different. Based on these hypotheses, a likelihood ratio is calculated [59]. A cluster can have a high trend either because it has a rate that is increasing faster than what is the case outside or because it has a rate that is decreasing less than the outside. Likewise, it can have a low trend if the opposite is true [59]. Such trends in our study could be spotted by the SVTT based on Poisson probability model that was used to identifying most likely clusters in higher and lower trend areas of TB and SPPTB. The SVTT scan statistic does not specifically identify clusters with high or low rates, but rather spots clusters with trends edging higher or lower compared to outside the cluster. Outcomes are noted in relation to the null hypothesis of all trends being the same [59]. SVTT can be specified to spot clusters with high trends only, low trends only or the simultaneous identification of both. The maximum windows size was adjusted to 25, 35 and 50% for both study area and time period. The output was collected in the format of descriptive tables and visualised by ArcGIS v.10.8.

### Software

Microsoft Excel 2016 was used for the descriptive analyses. ArcGIS v.10.8 (ESRI, Redlands, CA, USA), 2021), QGIS v.3.16.3 (free and open-source Geographic Information Systems) and SaTScan v.9.7 (software was developed by Martin Kulldorff together with Information Management Services Inc. Cambridge, Massachusetts, 2021) were used for spatio-temporal analyses.

## Results

### Descriptive results

**Fig. 2:**
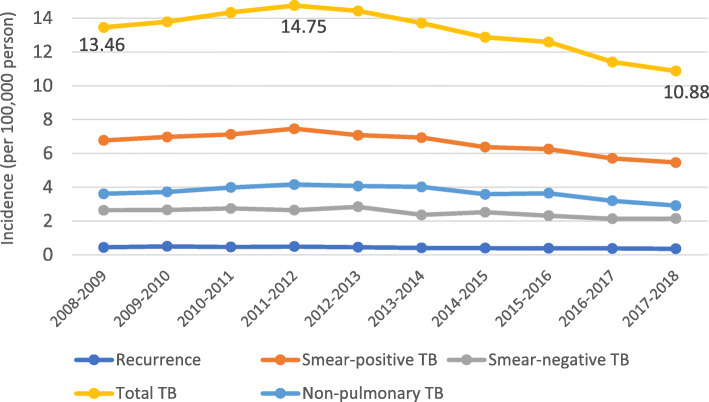
Tuberculosis incidence rate per 100,000 people in Iran 2008–2018

Table 1 shows the number and incidence of TB cases in Iran between 2008 and 2018 and Fig. 2 shows that the TB incidence per 100,000 people has decreased from 13.46 (95% CI: 13.19–13.73) in 2008 to 10.88 (95% CI:10.65–11.11) in 2018. The highest incidence of infection was due to SPPTB, non-pulmonary TB, and SNPTB.

**Table 1:** Incidence of tuberculosis patients in Iran between 2008 and 2018

Time	PTB+			PTB-			Non-pulmonary			Total			Number of Relapsed TB			Country Population
	Number of patients	Total incidence per 100,000	Mean (SD) among provinces	Number of patients	Total incidence per 100,000	Mean (SD) among provinces	Number of patients	Total incidence per 100,000	Mean (SD) among provinces	Number of patients	Total incidence per 100,000	Mean (SD) among provinces	Number of patients	Total incidence per 100,000	Mean (SD) among provinces	
March 2008–March 2009	4894	6.77	6.66 (5.61)	1907	2.64	2.71 (2.16)	2612	3.61	3.53 (2.05)	9733	13.46	13.30 (10.00)	320	0.44	0.39 (0.60)	72,326,307
March 2009–March 2010	5109	6.97	7.03 (6.03)	1944	2.65	2.69 (2.62)	2726	3.72	3.70 (2.06)	10,099	13.79	13.82 (10.76)	366	0.50	0.39 (0.53)	73,259,311
March 2010–March 2011	5286	7.12	6.93 (5.53)	2033	2.74	2.60 (2.16)	2954	3.98	3.84 (2.12)	10,639	14.34	13.83 (9.79)	343	0.46	0.46 (0.50)	74,204,360
March 2011–March 2012	5605	7.46	7.18 (5.79)	1987	2.64	2.49 (2.74)	3125	4.16	3.73 (2.25)	11,086	14.75	13.86 (10.82)	369	0.49	0.44 (0.49)	75,149,668
March 2012–March 2013	5386	7.08	6.93 (5.78)	2162	2.84	2.79 (2.63)	3096	4.07	3.78 (2.14)	10,987	14.43	13.95 (10.72)	343	0.45	0.44 (0.57)	76,124,602
March 2013–March 2014	5334	6.93	6.67 (5.42)	1816	2.36	2.38 (1.85)	3091	4.02	3.71 (2.19)	10,555	13.72	13.19 (9.5)	314	0.41	0.41 (0.47)	76,941,000
March 2014–March 2015	4975	6.38	6.14 (5.19)	1964	2.52	2.50 (2.15)	2795	3.58	3.39 (2.03)	10,044	12.87	12.41 (9.51)	310	0.40	0.37 (0.48)	78,028,486
March 2015–March 2016	4927	6.25	5.90 (4.81)	1823	2.31	2.30 (2.09)	2866	3.64	3.43 (2.02)	9917	12.59	12.00 (8.86)	301	0.38	0.38 (0.39)	78,771,605
March 2016–March 2017	4558	5.70	5.41 (4.42)	1701	2.13	2.04 (1.59)	2555	3.20	3.05 (1.52)	9118	11.41	10.86 (7.61)	304	0.38	0.35 (0.38)	79,926,269
March 2017–March 2018	4426	5.46	5.00 (4.16)	1741	2.15	2.14 (1.33)	2362	2.91	2.67 (1.75)	8819	10.88	10.13 (7.22)	290	0.36	0.32 (0.35)	81,069,998

*PTB+* Smear-positive pulmonary tuberculosis; *PTB-* Smear-negative pulmonary tuberculosis; *TB* Tuberculosis; *SD* Standard Deviation

**Fig. 3:**
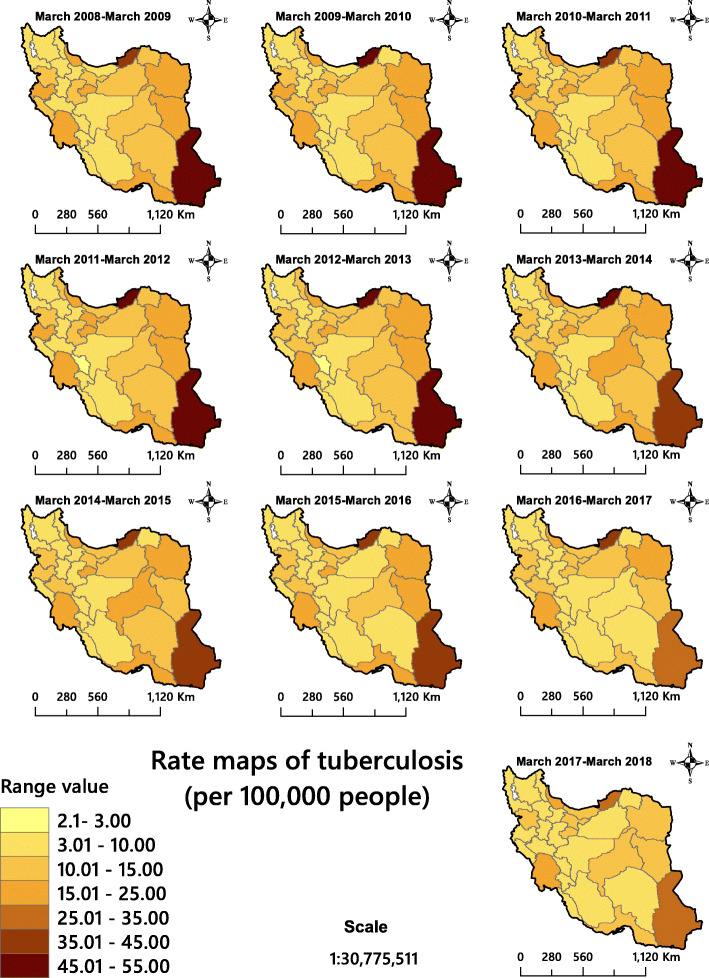
The total tuberculosis incidence in Iran 2008–2018 presented as descriptive maps. The figure has been created by free QGIS software

Figure 3 shows the descriptive map for total TB incidence cases in all the provinces. This figure shows an overview of the prevalence of TB, which is higher in eastern Iran. Sistan and Baluchestan in south-eastern Iran and the province of Golestan, which is located in northern Iran, had the highest incidence of TB among all provinces for all the study years. The figure also shows that the incidence rate of infection decreased gradually in the eastern provinces during 2008 and 2018.

### Results of spatial analyses

**Fig. 4:**
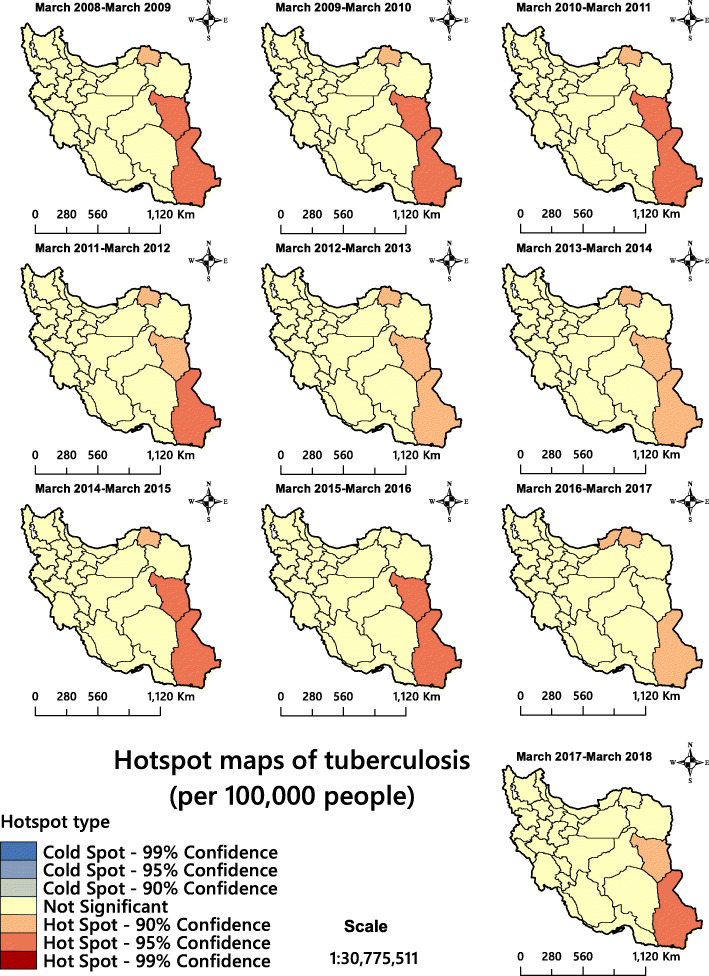
The total tuberculosis incidence in Iran 2008–2018 presented as space-time cluster maps. The figure has been created by free QGIS software

**Fig. 5:**
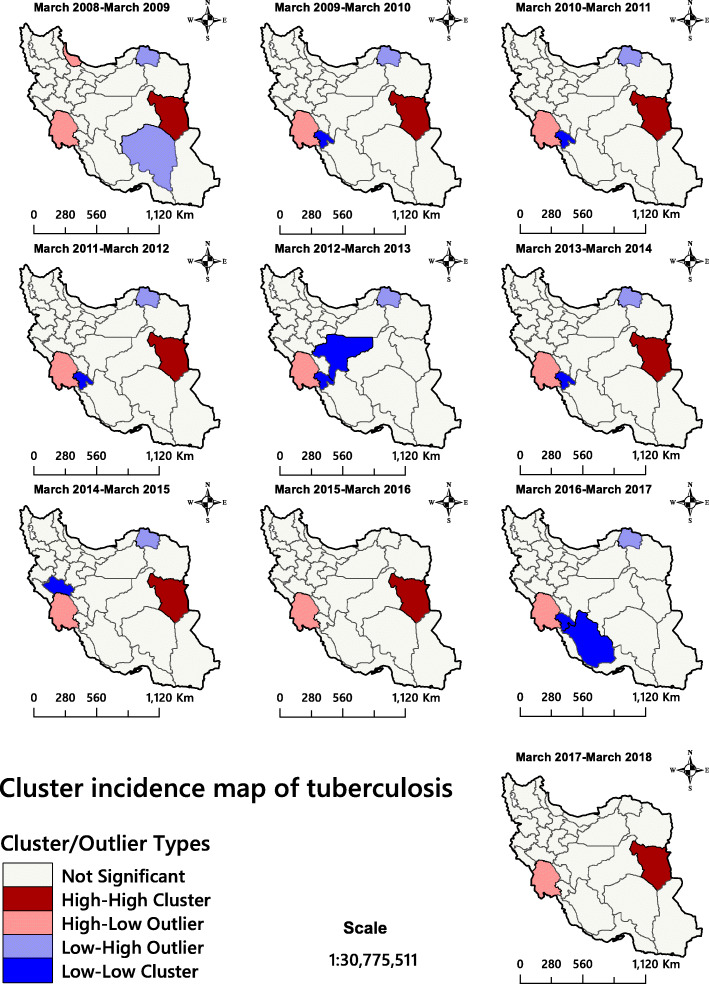
The total tuberculosis incidence in Iran 2008–2018 presented as cluster maps. The figure has been created by free QGIS software

The Getis-Ord Gi* statistic for total TB infectious identified north-eastern and south-eastern Iran as TB hotspots (Fig. 4). The analysis confirmed that the province of Sistan and Baluchestan and the province of Golestan had statistically significant higher rates of TB incidence than the rest of the country. Figure 5 shows the areas for total TB infectious clustering identified by Anselin’s Local Moran’s *I*. The analysis identified Khuzestan, a province in western Iran, as a high-low outlier throughout the 10 years covered by the study. This significant finding could not be obtained via the hotspot analysis because this approach only shows two kinds of clusters.

**Fig. 6:**
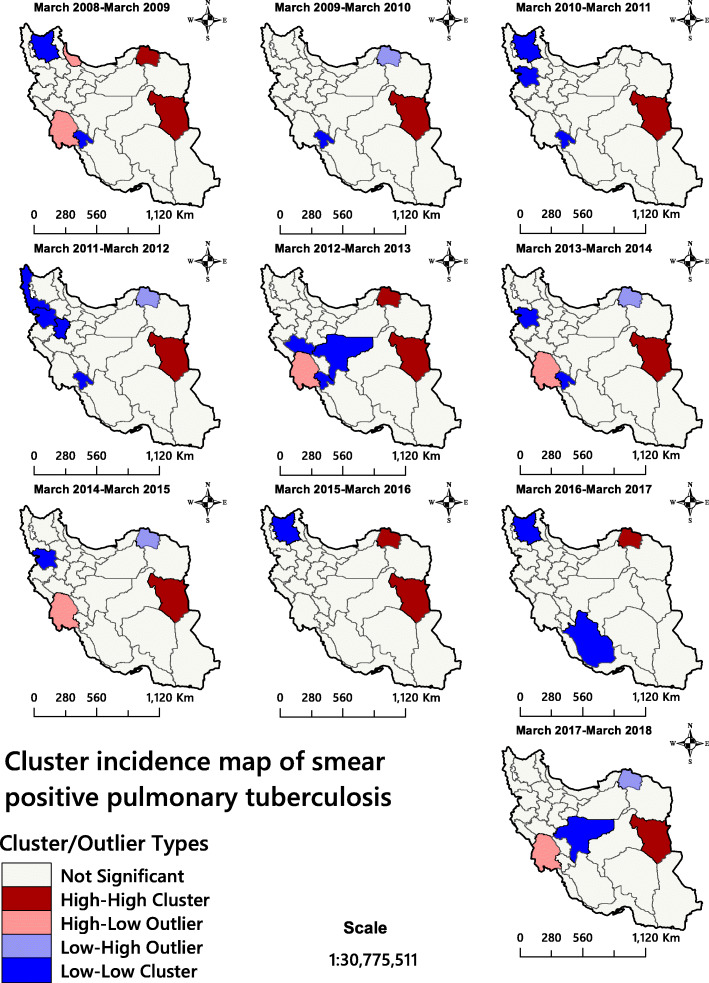
Smear-positive pulmonary tuberculosis incidence in Iran 2008–2018 presented as cluster maps. The figure has been created by free QGIS software

The province of Khuzestan was identified as a High-Low outlier of SPPTB cases for 5 years, while the province of Gilan was in this category for only 1 year (Fig. 6). In addition. it was noted that southern Khorasan Province was a High-High cluster for nine of the study years.

### Purely temporal analysis

Table 2 shows the results of a purely temporal analysis of total TB and SPPTB, with the three different maximum time window sizes. The results indicate that high-rate clusters of total TB were predominantly distributed in the time period between 2010 and 2014. Furthermore, low-rate clusters were observed in 2017 and 2018 (LLR = 303.99, *p* < 0.001) with all window sizes. A similar pattern was observed for SPPTB, except that very high-rate clusters were seen in 2011 and 2012 (LLR = 39, *p* < 0.001). In terms of areas with low rates, SPPTB clustering was distributed between 2015 and 2018, but were predominantly identified in 2017 and 2018 (at the 25% of the time window size).

**Table 2:** Purely temporal clusters identified by different window sizes, study period March-2008 to March-2018

Type of disease	Window size	Time frame	Number of cases	Expected cases	Annual cases / 100,000	OE	RR	LLR	*P*-value
Total TB	Areas with high rates								
	25%	***2012–2013***	22,073	20,425.02	14.2	1.08	1.10	81.73	0.001
	35%	*2011–2013*	32,712	30,226.28	14.2	1.08	1.12	143.69	0.001
	50%	*2010–2014*	53,366	49,895.66	14.0	1.07	1.15	238.58	0.001
	Areas with low rates								
	25%	***2017–2018***	17,937	21,059.13	11.2	0.85	0.82	303.99	0.001
	35%	*2017–2018*	17,937	21,059.13	11.2	0.85	0.82	303.99	0.001
	50%	*2017–2018*	17,937	21,059.13	11.2	0.85	0.82	303.99	0.001
Smear Positive Pulmonary TB	Areas with high rates								
	25%	***2011–2012***	10,891	10,089.69	7.1	1.08	1.10	39	0.001
	35%	*2011–2013*	16,277	15,113.59	7.1	1.08	1.11	63	0.001
	50%	*2010–2014*	26,720	24,948.57	7.0	1.07	1.15	124.32	0.001
	Areas with low rates								
	25%	***2017–2018***	8984	10,529.88	5.6	0.85	0.82	148.99	0.001
	35%	*2016–2018*	13,911	15,703.99	5.8	0.89	0.84	151.91	0.001
	50%	*2015–2018*	18,886	20,809.17	5.9	0.91	0.85	152.72	0.001

### Spatio-temporal analysis

**Fig. 7:**
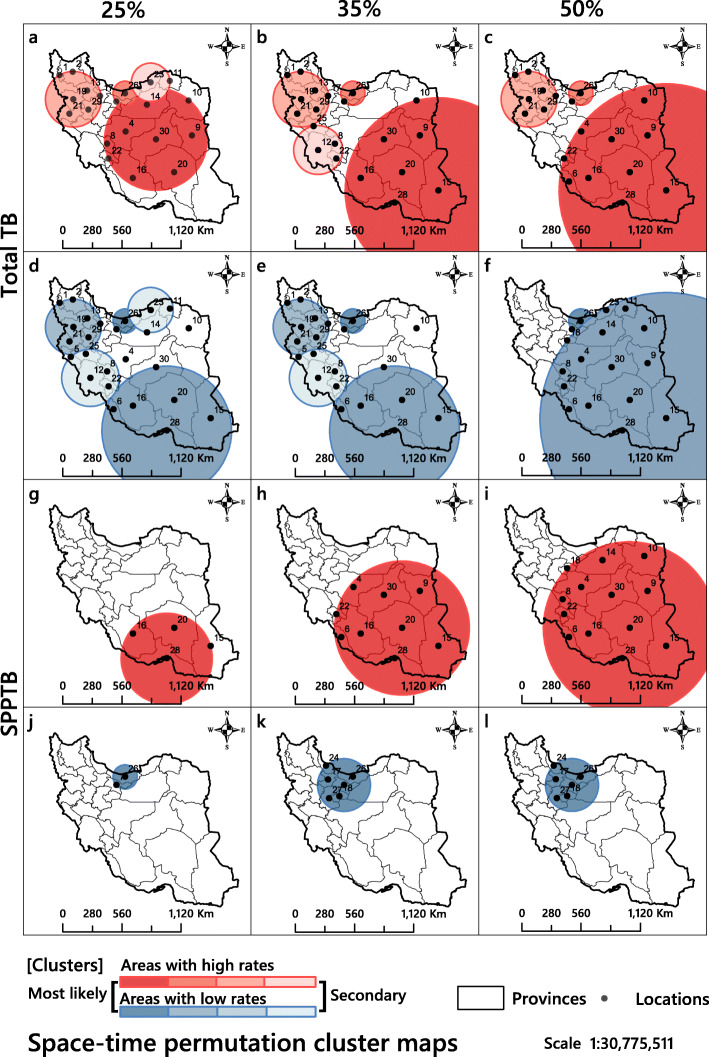
Spatiotemporal clustering of the incidence of total tuberculosis and smear-positive pulmonary tuberculosis in Iran 2008–2018. The results shown in columns 1 to 3 were obtained with the maximum windows of 25, 35 and 50%, respectively. The circles in red shades show locations with higher rates and circles in blue shades locations with lower rates. The figure has been created by free QGIS software

The statistically significant most likely high-rate total TB clusters were mainly distributed from centre to east in Iran (Fig. 7). Table 3 shows only the analysis for areas with high rates. Based on the 25% maximum window size, Isfahan, Chaharmahal and Bakhtiari, South Khorasan, Razavi Khorasan, Semnan, Fars, Kerman, Kohgiluyeh and Boyer-Ahmad and Yazd, altogether nine locations could be classified as high-risk areas (TS = 29.92, *p* < 0.05) (Fig. 7a). In all three windows of maximum clustering size, the years 2009 and 2010 were considered high-risk periods (Table 3). There were fewer clusters when we shifted the focus to SPPTB, however from the spatially point of view, they followed the same pattern as that of the total TB rates. In the SPPTB category, the years 2009 and 2010 were considered high-risk periods (Table 3), with most likely clusters located in the provinces Hormozgan, Kerman, Fars and Sistan and Baluchestan (TS =19.06, *p* < 0.05) (Fig. 7g).

**Table 3:** Most likely spatiotemporal TB and SPPTB high-rate clusters based on space-time permutation scan statistic model

Type of disease	Window size	Cluster name	Location IDs	Coordinates / radius	Time frame	Number of cases	Expected cases	OE	Test statistic	*P*-value
TB (total)	25%	1	**30, 4, 14, 9, 20, 16, 8, 10, 22**	(32.454000 N, 55.563900 E) / 484.96 km	**2009–2010**	5219	4692.67	1.11	**29.91**	0.000
		2	26, 7	(36.380600 N, 52.386100 E) / 113.98 km	2017–2018	4352	3891.38	1.12	27.33	0.00
		3	19, 21, 13, 29, 17, 2, 1	(35.683300 N, 46.983600 E) / 263.65 km	2009–2010	2497	2213.20	1.13	17.87	0.00
		4	23, 11	(37.315300 N, 55.082800 E) / 181.53 km	2013	1042	942.08	1.11	5.17	0.187
	35%	1	**15, 20, 28, 9, 30, 16, 10**	(27.919700 N, 60.724800 E) / 876.32 km	**2009–2010**	7285	6612.70	1.10	**35.47**	< 0.00
		2	26, 7	(36.380600 N, 52.386100 E) / 113.98 km	2017–2018	4352	3891.38	1.12	27.33	0.00
		3	19, 21, 13, 29, 17, 2, 1	(35.683300 N, 46.983600 E) / 263.65 km	2009–2010	2497	2213.20	1.13	17.87	0.00
		4	12, 8, 22, 25	(31.438500 N, 49.012100 E) / 231.32 km	2016–2018	3203	2988.46	1.07	7.75	0.013
	50%	1	**15, 20, 28, 9, 30, 16, 10, 6, 4, 22**	(27.919700 N, 60.724800 E) / 1008.53 km	**2009–2010**	8201	7451.95	1.10	**39.45**	< 0.00
		2	26, 7	(36.380600 N, 52.386100 E) / 113.98 km	2017–2018	4352	3891.38	1.12	27.33	0.00
		3	19, 21, 13, 29, 17, 2, 1	(35.683300 N, 46.983600 E) / 263.65 km	2009–2010	2497	2213.20	1.13	17.87	0.00
SPPTB	25%	1	**28, 20, 16, 15**	(27.031300 N, 56.495100 E) / 428.51 km	**2009–2010**	2362	2080.43	1.14	**19.06**	0.000
	35%	1	20, 28, 30, 15, 9, 16, 6, 4, 22	(29.615800 N, 57.296300 E) / 633.60 km	2009–2011	4514	4128.03	1.09	19.11	0.000
	50%	1	20, 28, 30, 15, 9, 16, 6, 4, 22, 8, 14, 10, 18	(29.615800 N, 57.296300 E) / 816.06 km	2009–2011	6948	6452.56	1.08	21.35	0.000

A cluster is statistically significant when its test statistic is greater than the critical value, which is, for significance level: Gumbel Critical Values: 0.00001: 13.533176 and 0.0001: 11.609444

In the case of areas with low clustering rates, in all three types of maximum clustering windows, several most likely and secondary clusters were seen to have formed. In terms of total TB areas with low rates, the most likely clusters formed in Mazandaran, Tehran and Alborz (TS = 93.03, *P* < 0.05) in all three windows (Fig. 7d,e,f). The low-rate areas were particularly clustered in the years 2009 and 2010 (Table 3). In terms of smear-positive pulmonary TB, a similar pattern was formed and locations 26 (Mazandaran) and 7 (Tehran and Alborz) were most likely clustered. But in smear-positive pulmonary TB type, in all three maximum clustering windows, only one main cluster was formed. The years 2009 and 2010 were times when low-rate areas were clustered more than any other years (TS = 40, *p* < 0.05). The spatio-temporal pattern of total TB was different from that of SPPTB. The low-rate total TB clusters, like the high-rate ones, stretched from the centre to the East of Iran, the SPPTB mainly formed in the northern parts of the country. Also, the secondary SPPTB clusters were not formed within the same area and time period.

### Spatial variation in temporal trends

**Fig. 8:**
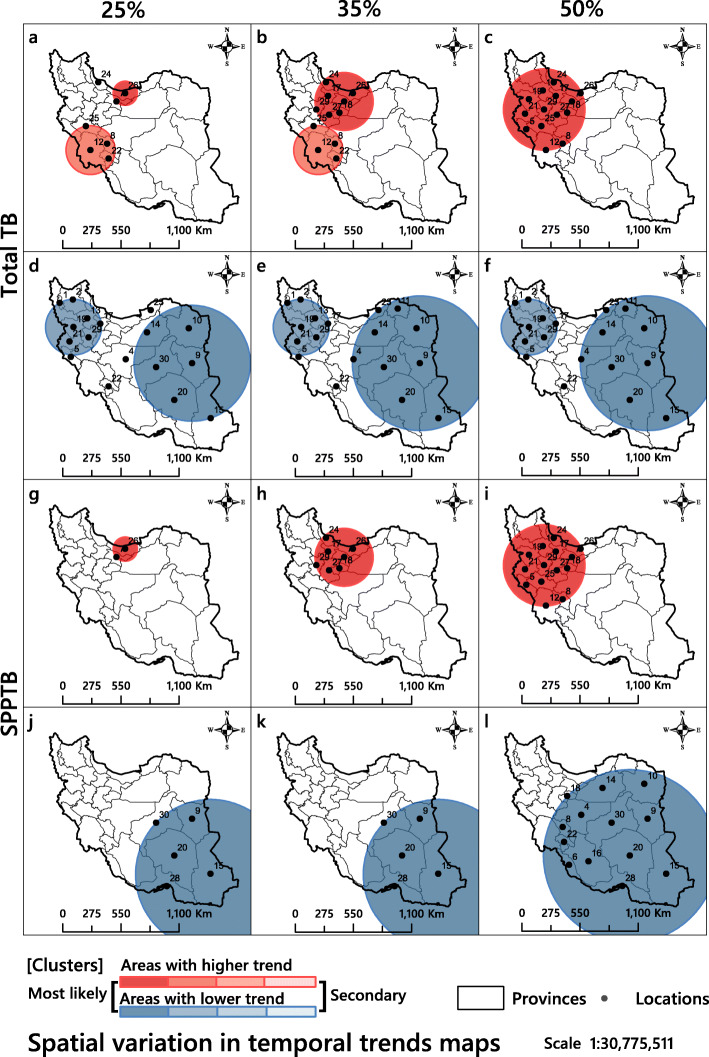
Spatial variations in temporal trends of the incidence rates of total tuberculosis and smear-positive pulmonary tuberculosis in Iran 2008–2018. The results shown in columns 1 to 3 were obtained with the maximum windows of 25, 35 and 50%, respectively. The circles in red shades show locations with increasing trends and circles in blue shades locations with lowering trends. The figure has been created by free QGIS software

Table 4 shows only the result of SVTT for areas with higher trends in all three window sizes (25, 35 and 50%). According to this table and Fig. 8, five high-trend clusters in the total TB category and three high-trend clusters in SPPTB category were found. The provinces Tehran, Alborz and Mazandaran were in high-trend statistically significant clusters in total TB (LRR = 62.05, *p*-value < 0.05) and SPPTB (LRR = 32.34, *p* < 0.05) (Table 4). In terms of areas with lower trends, statistically significant clusters were similarly formed in the different window sizes (Fig. 8). In the total TB category, for example with the 25% of window size, South Khorasan, Razavi Khorasan, Yazd, Kerman, Semnan, Sistan and Baluchestan, altogether six locations, were inside statistically significant clusters (LLR = 64.80, *p* < 0.05). In the SPPTB category, five locations included Sistan and Baluchestan, Kerman, Hormozgan, South Khorasan and Yazd (LLR = 44.38, *p* < 0.05). It was observed that incidence was decreasing overall but not in the same way in all areas.

**Table 4:** Spatio-temporal variations of TB and SPPTB in areas with higher trends, Iran, March 2008 to March 2018

Type of disease	Window size	Cluster name	Location IDs	Coordinates / radius	Population	Number of cases	Expected cases	Annual cases / 100,000	OE	RR	Inside time trend (annual decrease)	Outside time trend (annual decrease)	LLR	*P*-value
TB	25%	1	**26, 7**	(36.380600 N, 52.386100 E) / 113.98 km	18,063,139	21,911	23,660.62	12.1	0.93	0.91	0.130%	3.043%	**62.05**	0.001
		2	12, 8, 22, 25	(31.438500 N, 49.012100 E) / 231.32 km	7,960,272	10,836	10,427.04	13.6	1.04	1.04	0.471%	2.652%	19.51	0.001
		3	24	(37.253500 N, 49.490400 E) / 0 km	2,498,200	4834	3272.35	19.4	1.48	1.50	0.360%	2.549%	16.38	0.001
	35%	1	7, 26, 18, 17, 27, 24, 29	(35.666400 N, 51.475800 E) / 274.03 km	26,168,650	31,939	34,277.90	12.2	0.93	0.90	0.445%	3.323%	77.32	0.001
		2	12, 8, 22, 25	(31.438500 N, 49.012100 E) / 231.32 km	7,960,272	10,836	10,427.04	13.6	1.04	1.04	0.471%	2.652%	19.51	0.001
	50%	1	29, 27, 25, 17, 19, 13, 21, 18, 5, 7, 24, 8, 26, 12	(34.885200 N, 48.610600 E) / 384.83 km	38,534,499	47,909	50,475.74	12.4	0.95	0.90	0.891%	3.795%	91.30	0.001
SPPTB	25%	1	**26, 7**	(36.380600 N, 52.386100 E) / 113.98 km	18,063,139	10,434	11,830.66	5.8	0.88	0.85	0.083%	3.111%	**32.34**	0.001
	35%	1	7, 26, 18, 17, 27, 24, 29	(35.666400 N, 51.475800 E) / 274.03 km	26,168,650	15,652	17,139.46	6.0	0.91	0.87	0.487%	3.391%	38.96	0.001
	50%	1	29, 27, 25, 17, 19, 13, 21, 18, 5, 7, 24, 8, 26, 12	(34.885200 N, 48.610600 E) / 384.83 km	38,534,499	23,340	25,238.62	6.1	0.92	0.86	0.702%	4.035%	59.98	0.001

A cluster is statistically significant when its log likelihood ratio is greater than the critical value, which is, for significance level: (Gumbel Critical Values: 0.00001: 13.417707 and 0.0001: 11.182490)

## Discussions

This study provides an overview of the latest TB spatio-temporal epidemiological status in Iran. The results identified decreasing trends of TB during the period 2008–2018. The results are similar to that found by other studies in the last two decades [18, 19]. Despite the decreasing incidence of TB in Iran, there is still a need for screening, preventive, control, and therapeutic interventions, especially in high-risk areas such as western and eastern Iran.

Treatment at no cost, increased awareness of people, improved hygiene levels, the establishment of new TB research centres together with quicker and better access to TB diagnostics have all had a significant impact on TB reduction in Iran [18]. Importantly, the reduction of SPPTB was found to be particularly significant. Since TB is a communicable disease, early diagnosis and timely treatment can reduce its incidence, particularly of SPPTB, because it is transmitted to other people easier than the other types of TB [68]. We could observe this difference since we investigated both the total TB incidence rate and the SPPTB data. This analysis identified high-risk areas of SPPTB that would help to better targeting prevention endeavours. According to spatial variation in temporal trends (Fig. 8), it was observed that incidence is decreasing overall, but not in the same way everywhere. With slight changes in the number and distribution of space-time patterns, variations in areas with higher and lower trends in both total TB and SPPTB were almost homogeneous.

Global and Local Moran’s *I* statistics are powerful ways to detect spatial autocorrelation and purely spatial clusters [48, 53], but these methods alone are not able to identify hidden spatiotemporal patterns and clusters of diseases over time. For this reason, a modified method called Moran’s spatiotemporal autocorrelation statistic (MoransST) was proposed by Anderson and Ryan [69] to detect spatiotemporal patterns. Jaya et al. in two recent studies used MoransST to explore spatiotemporal clusters of Dengue disease in Indonesia [24, 31]. However, this methodology needs heavy mathematical calculations. Spatial scan statistics is another approach to identify spatiotemporal patterns. Kulldroff has provided a comprehensive package in SaTScan to run spatial scan statistics. Therefore, in this study, in addition to spatial analysis conducted by Local Moran’s *I* and Getis Ord G*, we used spatial scan statistics to perform purely temporal and spatio-temporal analyses. Scan statistics also accounts for multiple testing through the calculation of the highest likelihood of occurrence for all possible cluster locations and sizes [60, 66]. This feature allowed us to compare findings on a time-space scale and select the most desirable results as quickly as possible. The main important feature that makes SaTScan particularly suitable is that it can detect clusters of any size located anywhere in the study region [55, 60].

In recent years, geospatial analytical methods have been used as efficient tools to identify and visualise high-risk areas of infectious diseases [30, 70, 71]. Although the descriptive maps only visualise the TB incidence rate (Fig. 3), hotspot/cluster maps are needed for informed decision making (Figs. 4, 5 and 6). The latter maps reveal the statistically significant high-risk and low-risk areas with regard to TB incidence rate over space. In this study, the total TB cluster map showed that the province of Khuzestan was a High-Low outlier region in all 10 study years (Fig. 5), which means that the TB incidence rate in the province is high, while it is low in neighbouring areas. This is a remarkable finding that should urge policymakers to investigate this difference further. This result was not available through hotspot analysis, because it considers two kinds of clusters (high-risk and low-risk), and it cannot identify High-Low and Low-High areas (Fig. 4). The province of Khuzestan is on the Iran-Iraq border, so one reason could be the migration of Iraqi people to this province. Iraq is a high TB-burden neighbour of Iran, which has severely disrupted health services because of long internal wars [14, 18, 72]. Furthermore, the province has the highest average temperature in Iran and Ahvaz, the capital of Khuzestan, is in fact one of the hottest cities in the world [73]. This is of particular interest since Cao et al. (2016) found a positive relationship between temperature and TB incidence rate in a spatial-temporal epidemiology study in China [74]. A previous study [75] identified Al-Howizeh/Al-Azim marshes in Khuzestan and Sistan Basin as sand and dust storms sources in Iran. These sources are responsible for the dust storms in Khuzestan and Sistan and Baluchestan provinces. There is a clear correaltion between TB and dust storms [76]. These storms can cause the high rates of TB incidence in these areas. However, even if high temperature and sand storms could be reasons for this high TB incidence in our study, we lacked data to confirm this relationship, and further research is therefore required to explain the pattern observed.

The Getis-Ord Gi* statistic and spatio-temporal analysis of current study identified Sistan and Baluchestan and Golestan provinces as high risk areas for TB incidence. Assessing epidemiology of tuberculosis in recent years have reported Sistan and Baluchestan and Golestan as provinces with highest incidence rates in Iran [77, 78]. Golestan Province has a large number of immigrants from Sistan and Baluchestan. Most of TB patients in the province of Golestan inhabit in low socioeconomic and rural areas. Farm workers immigrated from Sistan and Baluchestan were identified as high-risk people [78]. Sistan and Baluchestan, called the center of TB in Iran, is neighbour to Afghanistan and Pakistan and many of Afghan immigrants live in this province. The high incidence of TB in Sistan and Baluchestan is expected because of long borders with Afghanistan [79, 80]. Also, extensive droughts and sandstorms are other factors which are responsible for high occurrence of TB in this area [80].

Based on the spatio-temporal clusters presented in this study, despite differences in the number and distribution of clusters in high-rate areas in different window sizes, the eastern and south-eastern parts of Iran were the focus of most likely cluster formations (Fig. 7). The eastern and south-eastern border neighbours of Iran are Afghanistan and Pakistan. These countries are main sources of TB, which affect TB transmission in Iran [18]. A spatio-temporal study of TB in Pakistan [32] revealed northern and western parts of country as high-risk clusters for TB. Western areas of this country share long borders with eastern and south-eastern Iran. Previous studies in Kerman [81], Hormozgan [82] and Semnan (Damghan) [83] provinces in Iran have confirmed the effect of immigrants, specially individuals who come from Afghanistan and Pakistan during TB occurrence. Afghan immigrants were known as high-risk people for TB occurrence and spread in Kerman Province [81]. A longitudinal study [84] assessed the TB pattern of immigrant populations by using Iranian TB register Program data for 2005–2011. This study reported that 97.1% of non-Iranian TB cases were Afghan immigrants [84]. Also, a previous study in Fars Province identified high incidence of TB in Afghan immigrants [85]. Another study [86] which investigated the trend of TB prevalence in Yazd City during 2005–2014, considered the referring of Afghan immigrants to health centres of Yazd for treatment as the cause of high TB rates in this province. A previous study reported high incidence of TB in Khorasan Province [87] and 26% of all diagnosed cases, during 5 years period, were foreign immigrants [88].

### Strengths and limitations

This is the first study using a comprehensive GIS-based approach to examine the spatio-temporal epidemiology of TB incidence rate in Iran as a whole. The study was conducted at the provincial level because the data was not available at finer geography scale in the whole country. As a result, the differences within the provinces were not examined. Previous studies demonstrated that sensitivity and specificity from individual studies were highly variable. Pooled results of the most widely used tests showed sensitivity at 76 and 59% and specificity at 92 and 91% in smear-positive and smear-negative patients, respectively [89]. Therefore, our results might be underestimated.

## Conclusion

For a resource-limited country, investigating the spatio-temporal patterns of TB incidence rate can be useful for regional policymakers knowledge regarding the high-risk provinces. Our findings provide strengthened evidence for the design of local interventions aimed at reducing and controlling TB incidence in high-risk areas by implementing geographically targeted prevention programmes and ensuring efficient allocation of resources. Further research is needed to assess the environmental and social factors in identified high-risk areas and to explore local and regional patterns of TB at small-area level. For example, we strongly recommend performing spatio-temporal studies to examine the TB incidence rate in the province of Khuzestan, located in the Iranian West to see whether there is any TB geographical variation inside the province.

## Recommendations

WHO Eastern Mediterranean region is one of the most prevalent areas of TB across the world. Twenty-two countries, including Iran, are located in this area in such a way that Pakistan has the highest amount of TB incidence, with 268 per 100,000 people among these countries, and the lowest amount is for the United Arab Emirates with 1 per 100,000 people in 2018 [90]. The countries of this area, including Iran, should examine passengers and immigrants with respect to TB from the high-risk neighbouring countries to reduce the incidence.

## Supplementary Information

**Additional file 1.**

## Data Availability

The dataset used and/or analysed during the current study is publicly available via supplementary file 1. The file contains all the raw data used for conducting the analyses.

## Abbreviations

TB: Tuberculosis
WHO: World Health Organization
SSPTB: Sputum Smear-Positive TB
SSNTB: Sputum Smear-Negative TB
GIS: Geographical Information Systems
HIV: Human Immunodeficiency Virus
MOHME: Ministry of Health and Medical Education
SPPTB: Smear-Positive Pulmonary Tuberculosis
SNPTB: Smear-Negative Pulmonary Tuberculosis
RR: Relative Risk
LLR: Log likelihood Ratio
SVTT: Spatial Variation in Temporal Trends
MoransST: Moran’s spatiotemporal autocorrelation statistic
